# Advances in biodiversity: metagenomics and the unveiling of biological dark matter

**DOI:** 10.1186/s40793-016-0180-8

**Published:** 2016-09-09

**Authors:** Robert J. Robbins, Leonard Krishtalka, John C. Wooley

**Affiliations:** 1 Biodiversity Institute, University of Kansas, 1345 Jayhawk Blvd., Lawrence, KS 66045 USA; 2 University of California, San Diego, USA

**Keywords:** Prokaryotes, Multi-cellular eukaryotes, Metagenomics, Biodiversity, Tree of life

## Abstract

**Background:**

Efforts to harmonize genomic data standards used by the biodiversity and metagenomic research communities have shown that prokaryotic data cannot be understood or represented in a traditional, classical biological context for conceptual reasons, not technical ones.

**Results:**

Biology, like physics, has a fundamental duality—the classical macroscale eukaryotic realm vs. the quantum microscale microbial realm—with the two realms differing profoundly, and counter-intuitively, from one another. Just as classical physics is emergent from and cannot explain the microscale realm of quantum physics, so classical biology is emergent from and cannot explain the microscale realm of prokaryotic life. Classical biology describes the familiar, macroscale realm of multi-cellular eukaryotic organisms, which constitute a highly derived and constrained evolutionary subset of the biosphere, unrepresentative of the vast, mostly unseen, microbial world of prokaryotic life that comprises at least half of the planet’s biomass and most of its genetic diversity. The two realms occupy fundamentally different mega-niches: eukaryotes interact primarily mechanically with the environment, prokaryotes primarily physiologically. Further, many foundational tenets of classical biology simply do not apply to prokaryotic biology.

**Conclusions:**

Classical genetics one held that genes, arranged on chromosomes like beads on a string, were the fundamental units of mutation, recombination, and heredity. Then, molecular analysis showed that there were no fundamental units, no beads, no string. Similarly, classical biology asserts that individual organisms and species are fundamental units of ecology, evolution, and biodiversity, composing an evolutionary history of objectively real, lineage-defined groups in a single-rooted tree of life. Now, metagenomic tools are forcing a recognition that there are no completely objective individuals, no unique lineages, and no one true tree. The newly revealed biosphere of microbial dark matter cannot be understood merely by extending the concepts and methods of eukaryotic macrobiology. The unveiling of biological dark matter is allowing us to see, for the first time, the diversity of the entire biosphere and, to paraphrase Darwin, is providing a new view of life. Advancing and understanding that view will require major revisions to some of the most fundamental concepts and theories in biology.

## Background

This paper grew out of our participation in an NSF-funded effort to harmonize data standards used by the biodiversity research community with those being developed and used in the genomic research community and, in particular, with the emerging and growing metagenomic research community. Although integrating genomic data from multicellular eukaryotes into biodiversity data sets was straightforward, dealing with metagenomic data in a traditional biodiversity context proved to be difficult for conceptual, not technical, reasons.

Because scientific data models should correspond to the reality being modeled, good models usually contain data objects that are formal abstractions of fundamental scientific concepts. When new discoveries challenge these fundamental concepts, the underlying data models must adapt or become obsolete. For example, the discovery that a gene could occur within another gene [1] rendered obsolete any data model that was based on the fundamental assumption that genes are non-overlapping objects.

Although non-eukaryotic microbes have long been known to be a ubiquitous and substantial component of the biosphere, the vast majority have been unculturable and thus completely resistant to study, rendering them effectively invisible. Until recently, a microscopic examination of any environment revealed a teeming, but largely mysterious prokaryotic world. (Note: Although “prokaryote” has been deprecated as a taxonomic concept [2], in this document we use the word in the informal sense of “not a eukaryote.”) A 1997 colloquium report from The American Academy of Microbiology [3] noted:

[I]n contrast to plants and vertebrate animals in which 85 to 90 % have been described, it is conservatively estimated that less than 1 % of the bacterial species … are currently known.

In 1998, however, a new technology began to emerge that would transform our ability to study prokaryotes: Handelsman et al. [4] coined the term “metagenome” to characterize the collective genomes of all microbes in a soil sample. They proposed to analyze this soil metagenome directly, without first having to culture the organisms in the soil. Over the next five years a handful of papers explored the possibilities of this new method, which (thanks to the constant drop in cost of sequencing) moved from creating clone libraries to the generation of full sequences. In 2003, Schloss and Handelsman [5] offered the term “metagenomics” to describe “the genomic analysis of uncultured microorganisms.”

As the utility of metagenomics became apparent, its use grew rapidly. A PubMed query shows that the number of published papers involving metagenomes and metagenomics has grown from one in 1998 to nearly ten thousand today. Metagenomics has become almost a new kind of instrument—a sort of “genomoscope”—that allows researchers to *see* the prokaryotic world for the first time. The view is stunning. It is essentially Humboldt in a new biosphere (cf., [6, 7]): wherever the genomoscope is pointed, new discoveries swarm into view.

These discoveries are showing that the full biosphere is substantially more complex than previously conceived and that a conception of nature based only on the biology of multicellular eukaryotes (MCEs) is decidedly inadequate. Attempting to understand and describe the entire biosphere using concepts based only on the biology of MCEs is like trying to understand all vertebrates by studying only hummingbirds.

Furthermore, it has long been known that every multicellular organism coexists with large prokaryotic ecosystems—microbiomes—that completely cover its surfaces, external and internal. Recent studies have shown that these associated microbiomes are not mere contamination, but instead have profound effects upon the function and fitness of the multicellular organism. We now know that all MCEs are actually functional composites, holobionts, composed of more prokaryotic cells than eukaryotic cells and expressing more prokaryotic genes than eukaryotic genes. A full understanding of the biology of “individual” eukaryotes will now depend on an understanding of their associated microbiomes.

In 1908, William Bateson [8] described the significance of the emerging new methods of Mendelian genetics:

In research, as in all business of exploration, the stirring times come when a fresh region is suddenly unlocked by the discovery of a new key. Then conquest is easy and there are prizes for all. … It is no hyperbolical figure that I use when I speak of Mendelian discovery leading us into a new world, the very existence of which was unsuspected before.

As genetics did for the 20^th^ century, so metagenomics is doing for the 21^st^ century—it is leading us into a new world of discovery, with prizes for all. Applied to the biosphere, two genomoscope discoveries are fundamental: first, most of the world’s biodiversity occurs in the prokaryotic realm, the full nature of which was unsuspected before. Second, many “foundational” biological concepts—such as the objective reality of individual organisms—are inadequate to describe and explain this emerging new world. The full incorporation of the prokaryotic realm into our understanding of the biosphere will enrich biology, but at the expense of our having to rethink and reconceptualize some of our most basic notions.

The resulting conceptual adjustments will offer real challenges to our current view of biodiversity and will greatly complicate the informatics tools needed to document biodiversity. Not only will biodiversity informatics need to deal with an explosion in the amount of biodiversity-relevant data, it may well need to accommodate data that are of a conceptually different form. As any informatics professional knows, making changes to an underlying data model is always difficult and fraught with risk. Making changes to conceptual base classes is the hardest of all. This will be the world of 21^st^ Century biodiversity informatics.

## The quantum-classical duality in biology

Steven Weinberg elegantly captured the duality between classical physics and the new quantum cosmology: “Our expanding universe is mostly dark energy and dark matter. In this darkness there is a small admixture, a few percent of the whole, which consists of the ordinary matter that makes up the stars and planets and us” [9].

Analogously, classical biology describes the familiar world of multi-cellular eukaryotes (MCEs), which historically were believed to *be* the biosphere. New tools are now making it possible to study the vast, heretofore mostly uncharacterized, prokaryotic world of microbial life. Biological *dark matter* [10], akin to cosmological dark matter, is an apt descriptor for this overwhelming and unseen component of the biosphere. These studies are forcing us to recognize that, rather than typifying life, MCEs are actually a derived and highly constrained evolutionary subset of the biosphere.

Prokaryotes comprise about half the biomass of the non-viral biosphere [11] and nearly all of its physiological biodiversity (defined in terms of diversity of tolerated ecological conditions and biochemical capacities). To paraphrase Weinberg, our biosphere is mostly biological dark matter. In this darkness there is a small admixture, a few percent of the whole, which consists of the ordinary multi-cellular eukaryotic life that makes up the fungi and plants and animals and us. As such, the properties and behavior of biological dark matter comprise what might be termed, metaphorically, quantum biology.

The relationship of quantum biology to classical biology has much in common with the relationship between quantum and classical physics. In both cases, the quantum realms deal with very small entities that behave in a counter-intuitive manner, profoundly different from classical understanding. And, in both cases the quantum realm is proving to be the more fundamental, with the classical view describing a derived and specialized condition.

Despite MCEs being merely that “few percent of the whole,” nearly all of classical biology, from Aristotle onwards, has been based on the composition and characteristics of MCEs. To put it bluntly, classical biology has been the study of MCEs by MCEs for MCEs.

Woese and Fox [12], in perhaps the most important biodiversity discovery of the past 100 years, used what were then cutting edge molecular tools to disprove the existing notion that the biosphere could be divided dichotomously into eukaryotes and prokaryotes. Instead, hidden among the prokaryotes they found another group—the Archaea—that was as different from the bacteria as either were from the eukaryotes.

These two ideas proved revolutionary: (1) that molecular tools were ideally suited for biodiversity research; and (2) that, based on that research, the biosphere contained three fundamentally different forms of life—Archaea, Bacteria, and Eukarya. Woese initially referred to his new categories as kingdoms, or urkingdoms, but he later proposed the new concept of *domain* [13].

Not surprisingly, Woese’s work was originally doubted, even ridiculed, by many biologists. One referred to him as “a crank, who was using a crazy technique to answer an impossible question” [14]. Subsequent improvements in sequencing technology, however, allowed the confirmation of a three-domain biosphere, of which the two prokaryotic realms are essentially alien to classical biology. Now, the three-domain view is well established, even at the level of introductory textbooks.

## One biosphere, two mega-niches

It is well known that relative size greatly affects *how* organisms interact with the world. A substantial collection of papers and monographs have characterized the effects of size on biological form and function (e.g., [15–17]), with emphasis on differing mechanical interactions with the environment, as, for example, elegantly put by Haldane [18]:

To the mouse and any smaller animal (gravity) presents practically no dangers. You can drop a mouse down a thousand-yard mine shaft; and, on arriving at the bottom, it gets a slight shock and walks away, provided that the ground is fairly soft. A rat is killed, a man is broken, a horse splashes. For the resistance presented to movement by the air is proportional to the surface of the moving object. Divide an animal’s length, breadth, and height each by ten; its weight is reduced to a thousandth, but its surface only to a hundredth. So the resistance to falling in the case of the small animal is relatively ten times greater than the driving force. An insect, therefore, is not afraid of gravity; it can fall without danger, and can cling to the ceiling with remarkably little trouble. It can go in for elegant and fantastic forms of support like that of the daddy-longlegs. But there is a force which is as formidable to an insect as gravitation to a mammal. This is surface tension. A man coming out of a bath carries with him a film of water of about one-fiftieth of an inch in thickness. This weighs roughly a pound. A wet mouse has to carry about its own weight of water. A wet fly has to lift many times its own weight and, as everyone knows, a fly once wetted by water or any other liquid is in a very serious position indeed. An insect going for a drink is in as great danger as a man leaning out over a precipice in search of food. If it once falls into the grip of the surface tension of the water—that is to say, gets wet—it is likely to remain so until it drowns.

Less well known, at least among biologists, is that at sufficiently small sizes, mechanical interaction with the environment becomes difficult and then virtually impossible. In fluid dynamics, an important dimensionless parameter is the Reynolds Number (abbreviated *Re*), which is the ratio of inertial to viscous forces affecting the movement of objects in a fluid medium (or the movement of a fluid in a pipe). Since *Re* is determined mainly by the size of the object (pipe) and the properties (density and viscosity) of the fluid, organisms of different sizes exhibit significantly different *Re* values when moving through air or water (Table 1) [19].

**Table 1:** Reynolds numbers exhibited by various organisms (after [19])

Organism, moving in fluid medium	Reynolds number
A large whale, swimming at 10 m/s	300,000,000
A tuna also swimming at 10 m/s	30,000,000
A duck, flying at 20 m/s	300,000
A large dragonfly, going 7 m/s	30,000
A copepod in a speed burst of 0.2 m/s	300
Flapping wings of the smallest flying insects	30
An invertebrate larva, 0.3 mm long, at 1 mm/s	0.3
A sea urchin sperm at 0.2 mm/s	0.03
A bacterium, “swimming” at 0.01 mm/s	0.00001

A fish, swimming at a high ratio of inertial to viscous forces, gives a flick of its tail and then glides for several body lengths. A bacterium, “swimming” in an environment dominated by viscosity, possesses virtually no inertia. When the bacterium stops moving its flagellum, the bacterium “coasts” for about a half of a microsecond, coming to a stop in a distance less than a tenth the diameter of a hydrogen atom. Similarly, the movement of molecules (nutrients toward, wastes away) in the vicinity of a bacterium is dominated by diffusion. Effective stirring—the generation of bulk flow through mechanical means—is impossible at very low *Re* [20].

Although prokaryotes can and do move, movement at very low *Re* differs profoundly from movement as experienced by humans. Collectively, the effects of low *Re* are such that a bacterium cannot move *through* a liquid medium at all, at least not in the sense that a fish moves through water. When a bacterium does “swim,” it carries with it a large surrounding bubble of local fluid, substantially greater than its own mass, so that the actual arrival of nutrient molecules at the bacterium’s surface is governed by the rate of diffusion through that local bubble, unaffected by the movement of the bacterium and its bubble through the fluid medium [20]. In a world dominated by viscosity and diffusion it is almost impossible to gain mechanical advantage through morphological change. Consequently, there is relatively little morphological differentiation among groups of prokaryotes.

Nearly all MCEs operate with moderate to high *Re* values, where mechanism matters. As a result, MCEs differ from the two prokaryote domains both in primary biological function (e.g., nucleated vs. non-nucleated cells) and also in occupying a different, unique ecological mega-niche: MCEs interact *mechanically* with the environment, whereas prokaryotes interact *physiologically*. Indeed, this MCE-prokaryote mega-niche dichotomy is reflected in the nature of their biodiversity. The bulk of MCE biodiversity, from red algae to redwood trees and from ciliates to cetaceans, is morphological, whereas the bulk of prokaryote biodiversity is physiological, operating across much larger environmental ranges of temperatures and chemistries than imaginable for MCEs.

Of course, MCEs exhibit some physiological diversity, just as prokaryotes exhibit some morphological diversity, but MCEs exhibit much greater morphological diversity than prokaryotes and prokaryotes show much greater physiological diversity than MCEs. For example, prokaryotes have been found living in temperatures ranging from −2° to +121 °C and in pH values from 3.0 to 11.0. On the other hand, all mammals have a core body temperature of 38 ± 2.0 °C, despite their vast morphological diversity. To be sure, some mammals live in environments with temperatures substantially below freezing, but they do this using morphological (i.e., mechanical) adaptations that allow the maintenance of the standard mammalian core metabolic temperature, despite extreme external temperatures. Indeed, much of what passes for MCE physiological variation is actually mechanical variation, evolutionarily contrived to allow similar physiologies to operate in dissimilar environments.

## Classical biology vs. Dark matter biology

Although the existence and ubiquity of microscopic life has been known since the seventeenth-century work of van Leeuwenhoek, efforts to understand the biology of the very small were frustrated by the absence of appropriate tools and methods, especially for prokaryotes. For example, studies have shown that when cell counts of environmental prokaryotic samples were assessed by direct observation vs. the production of colonies, a *plate-count anomaly* occurred. Historically, far more cells have been seen by direct observation than could not be enticed to develop into colonies [21]. Measurements of the ratio of culturable to actual cells range from 10^−2^ to 10^-8^, indicating that the vast majority of environmental prokaryotic cells cannot be studied in the laboratory, rendering them—until now—effectively invisible to science.

Being intractable to research, prokaryotic life became a kind of biological dark matter—life forms known to be present and abundant, but whose properties and behavior could not be assessed. The analogy to cosmological dark matter is obvious. Now, the emergence of metagenomic analytical methods [22] has rendered visible this heretofore hidden half of the biosphere—at least as genomic avatars. The results are dazzling.

Most profound is the demonstration that many fundamental notions in classical biology in fact only apply to the MCE realm, *viz*: (a) individual organisms are objectively real, fundamental units of the biosphere, (b) within cells, the *content* of the genome is extremely stable, protected, and highly regulated, (c) barring mutation, genetic novelty is acquired only during reproduction, largely as the result of recombinations generated during sexual reproduction, (d) all life can be organized into similarly defined species, and (e) with perfect knowledge, the biosphere could be arranged into one true, unified tree of life.

As detailed below, none of these foundational notions of classical biology can be applied to the biology of prokaryotes. Furthermore, compared with MCEs, prokaryotes operate in a spatio-temporal context that is quantitatively so different as to be almost qualitatively incommensurate.

### Growth vs. reproduction: the notion of the individual

In MCEs, a distinction is made between *growth*—cell divisions that simply result in an increase in size of an individual; and *reproduction*—cell divisions and fusions that result in the creation of a single, genetically novel cell, the zygote, that will be the founding cell for a new individual). Mitosis drives growth by pro-ducing daughter cells with (barring mutation) genetic content identical to each other and to the parental cell. Meiosis allows reproduction by reducing the genetic content of gametes so that the combination of two gametes into a zygote reconstitutes the standard genetic complement. With recombination, meiosis also adds to genetic novelty, at least at the level of combinations of genetic elements.

**Fig. 1:**
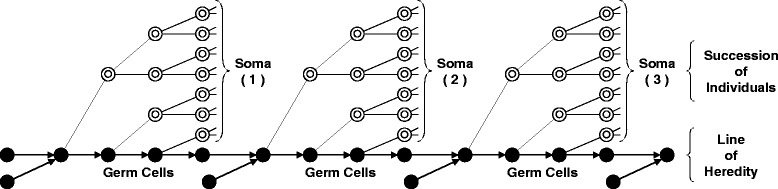
The separation somatic and germ cell lines as necessitated by sexual reproduction and as described by Weismann [23] in his germplasm theory. This model rules out the inheritance of characteristics acquired by an individual’s somatic tissue, as there is no route for hereditary material to move from somatic cells into the germ line

Shortly after mitosis and meiosis had been described, Weismann [23] recognized that the handling of the hereditary material in the cell-division processes necessary for sexual reproduction meant that all of the cells in an individual could be divided into *somatic* cells—cells that make up the body, but that will not lead to gametes; and *germ-line* cells—the cell lineage within the individual that will lead to gametes. In the Weismannian view, “individuals” are large aggregations of physically connected, genetically identical somatic cells that carry a genetic payload sequestered in the germ line. Somatic cells, and thus individuals, are mortal, whereas cells in the germ line are potentially immortal (Fig. 1).

In the dark-matter realm of prokaryotes, none of this is true. Prokaryotes have only one kind of cell division and thus, for them, there can be no distinction between growth and reproduction. With no distinction between somatic cells and germs cells, individuals in the Weismannian sense cannot occur.

### Enforced stability of genomic content

Within MCE cells, genetic information is stored in a very stable, heavily regulated genome whose content is well protected from outside influences. Generating a multi-cellular state requires a regulated genome [24, 25], and the successful regulation of gene expression, both in development and in physiology, depends in part on the maintenance of stable genomic content. Indeed, maintaining stability of genomic content is such an essential attribute of MCE biology that, among classical biologists, “genomic instability” is generally regarded as a condition related only to pathology. For example, a PubMed search on “genomic instability” returns more than 15,000 papers, nearly all dealing with cancer and other pathologies. Yet, as discussed below, rigorous enforced stability of genomic content does not occur in prokaryotes.

Among MCEs, when genetic or genomic changes produce significant adverse effect, many of the resulting problems are produced by perturbations in development, or disruptions of a complicated bit of physiology necessary to accomplish some critical function in differentiated somatic tissue. For MCEs, especially animals, quantitative balance among genomic components is so important that polyploidy always has detectable effects and aneuploidy is almost always significantly disruptive.

In humans, an individual with an extra copy of chromosome 21 (that is, an extra 0.75 % of the diploid human genome) exhibits significant deleterious effects. Larger aneuploidies produce profoundly deleterious effects and human aneuploidies involving 5 % or more of the genome are almost always lethal *in utero*. Among prokaryotes, on the other hand, fully functional cells of the same “species” may vary by 80 % or more of their genetic content.

The enforced stability of genomic content in MCEs is necessary for maintaining a non-pathological multi-cellular, differentiated condition. Curiously, this critical attribute is not on any of the lists of essential eukaryotic pre-adaptations necessary for the evolution of multi-cellularity. This absence may be a result of the pervasive MCE-centricity of much classical biology thinking. That is, this enforced stability of genomic content is such an essential component of MCE biology that MCE-oriented biologists may have mistaken it for an essential requirement of life, rather than as a derived requirement specific to the MCE differentiated state.

Perhaps the defining evolutionary aspect of eukaryotic biology—the endosymbiotic domestication of a bacterium to become the mitochondrion—required the development (or the pre-existence) of mechanisms for enforcing the stability of genomic content. Since exploring that idea is outside the scope of this paper, here we merely note that, rather than being an essential attribute of life, enforced stability of genomic content is yet another example of MCE atypicality.

### The prokaryotic pan-genome and horizontal gene transfer

#### The pan-genome

Although the enforced stability of genomic content is ubiquitous among MCEs, the opposite is proving to be the case among prokaryotes, which exhibit remarkable and adaptive plasticity of genomic content. Early bacterial whole-genome sequencing efforts discovered that whenever a particular “species” was re-sequenced, new genes were found that had not been detected earlier—entirely new genes, not merely new alleles. This led to the concepts of the bacterial *core-genome,* the set of genes found in all members of a particular “species”, and the *flex-genome*, the set of genes found in some, but not all members of the “species”. Together these make up the species’ *pan-genome* [26–28]. In many cases, a typical individual bacterial cell carries less than 50 % of the genes found in the species’ pan-genome—a level of genomic plasticity that in MCEs is only seen in highly unregulated tumors.

In one classic study, a genome comparison of three pathogenic *E. coli* strains found that “only 39.2 % of their combined (nonredundant) set of proteins actually are common to all three strains” [29]. Another study involving full sequences for 61 different *E. coli* and *Shigella* spp. strains [30] produced even more striking results.

The predicted pan-genome comprises 15,741 gene families, and only 993 (6 %) of the families are represented in every genome, comprising the core genome. The variable or ‘accessory’ genes thus make up more than 90 % of the pan-genome and about 80 % of a typical genome; some of these variable genes tend to be co-localized on genomic islands. The diversity within the species *E. coli*, and the overlap in gene content between this and related species, suggests a continuum rather than sharp species borders in this group of *Enterobacteriaceae*.

This is a stunning amount of variation within one “species” of bacteria. (Strains in the genus *Shigella* are now regarded as *E. coli* variants that need reclassification [30]). If only strains currently named *E. coli* are considered, the number of core gene families rises from 993 to 1472, a slight increase of 6 % to 9 % of the total pan-genome. Some pairwise comparison of two *E. coli* strains may show as little as 20–25 % overlap in gene content (the 9 % common core genes, plus 11 % shared flex genes). By comparison, current data suggest a 30 % overlap in gene content between humans and mice. That is, there may be more genetic similarity between a randomly selected human and a randomly selected mouse than there is between two bacterial cells of the same “species.” Clearly, a species concept that encompasses that much genetic variation is not compatible with the species concept as generally applied to MCEs.

Maps of the 61 genomes revealed that the flex genes occurred in clusters on gene islands, not randomly dispersed across the genome. Substantial differences in genome size were also observed. The strain with the largest genome (*E. coli* O157:H7) contained 5.7 million base pairs of DNA, whereas the smallest (*E. coli* BL21) contained only 4.56 million base pairs. *E. coli* seems to have an open and apparently unbounded pan-genome. That is, as the numbers of sequenced *E. coli* strains continues to grow, the number of discovered core genes remains about the same, while the number of flex genes grows linearly with the number of strains sequenced. Land et al. ([31], p. 141; see also Figure 6, p. 150) asserted that comparison of more than 2000 *Escherichia coli* genomes finds an *E. coli* core genome of about 3100 gene families and a total of about 89,000 gene families.

More importantly, these differences in gene content and genome size were far from neutral. K-12 and B strains of *E. coli* are harmless commensals, routinely found in the gut of all homeothermic animals. O157:H7, on the other hand, is a dangerous pathogen, causing potentially fatal hemorrhagic diarrhea in infected humans.

#### Horizontal gene transfer

The pathology-inducing genes of O157:H7 appear to have been acquired, likely via prophage, by a non-pathogenic *E. coli* ancestor, perhaps 20,000 years ago. That is, horizontal gene transfer (HGT) can lead to the profound phenotypic change from benign commensal to lethal pathogen. “Horizontal” in this context refers to the lateral or “sideways” movement of genes between microbes via mechanisms not directly associated with reproduction. HGT among prokaryotes can occur between members of the same “species” as well as between microbes separated by vast taxonomic distances. As such, much prokaryotic genetic diversity is both created and sustained by high levels of HGT [32]. Although HGT can occur for genes in the core-genome component of a pan-genome, it occurs much more frequently among genes in the optional, flex-genome component.

In some cases, HGT has become so common that it is possible to think of some “floating” genes more as attributes of the environment in which they are useful rather than as attributes of any individual bacterium or strain or “species” that happens to carry them. For example, bacterial plasmids that occur in hospitals are capable of conferring pathogenicity on any bacterium that successfully takes them up. This kind of genetic exchange can occur between widely unrelated taxa.

Also, HGT between dietary bacteria and gut microbes can lead to the acquisition of new dietary capabilities by their hosts without requiring a change in the “species” composition of the gut microbiome. For example, humans in Japan possess gut microbiomes that are similar to those found in North Americans, where “similar” means “possessing the same species composition, as measured by rRNA analysis.” However, unlike North American gut microbiomes, Japanese gut microbiomes can digest seaweed because of genes acquired through HGT from a marine microbe (*Zobellia galactanivorans*) that provide porphyranases, agarases and associated proteins useful in the digestion of porphyran from marine red algae [33].

### Transmission vs. acquisition genetics

In classical MCE biology, organisms acquire new hereditary material precisely once, when genetic material is transmitted from parent to progeny during the formation of the zygote. This produces a genetically novel cell that will form the clonal basis for the new Weismannian individual to come. Following the rediscovery of Mendel’s work, the study of these processes came to be known as *transmission genetics*, thereby linguistically capturing the implicit notion that, barring rare mutation, a new individual acquires new genetic material only through the active transmission of that material across generations (of individuals) via the cellular processes of meiosis, gametogenesis, and fertilization.

Prokaryotes, like MCE’s, exhibit transmission genetics in receiving genes transmitted from a parental cell at the moment of cell division. Because this involves the simple replication of the existing genetic material, from the perspective of MCE biology it would seem that prokaryotes should possess very little genetic variability, leading one author of a widely used text on biodiversity to assert:

Essentially, every prokaryote is its own lineage, either dying, budding off, or splitting into daughter cells that are clones of the parent, in the process of asexual reproduction. … Daughter cells are clones, with the same DNA as the parent cell, so they are already well adapted to the microenvironment. Prokaryotes gamble against a change in the environment: if a change occurs that kills an individual, that change will most likely wipe out all that individual’s clones too. Prokaryotes have no way to affect the future of their genes. They can only pass them on unchanged to their offspring. … In organisms that reproduce by cloning, a favorable mutation can spread successfully over many cycles of cloning if it occurred in an individual that divided faster than its competitors. The environment selects or rejects the whole DNA package of the mutant individual, which either divides or dies. This is a one-shot chance, and many potentially successful mutations may be lost because they occur in an individual whose other characters are poorly adapted ([34], pp. 34–35).

This MCE-centric viewpoint errs in completely omitting the role of HGT—*acquisition genetics*—in generating vast genetic variation among prokaryotes—much more than transmission genetics does in MCEs. Prokaryotes do not generate genetic *variability* through transmission genetics, but they acquire genetic novelty through acquisition genetics—the acquisition of genes directly from the environment via mechanisms not involving reproduction. Routine acquisition genetics, uncoupled from reproduction, simply does not occur among MCEs. Prokaryotic acquisition genetics provides virtually unlimited opportunities for genetic variance. One study [35] carried out a full genomic assessment of several hundred individual marine bacteria (*Vibrio splendidus*) collected at the same location and found essentially no two alike. The potential implications of widespread acquisition genetics via HGT for an understanding of evolution are profound [36].

Furthermore, we know that the ability to acquire exogenous DNA (known as *competence*) can be adjusted by bacteria in response to environmental conditions, with competence generally increasing under conditions of stress [37]. The similarity to the pattern seen in some MCEs, such as aphids, that alternate between parthenogenetic and sexual reproduction is suggestive: both reproduce asexually in benign conditions, switching to a genetic-diversity-generating mechanism when conditions are harsh. It has also been shown that HGT is more likely to occur between strains of bacteria that possess the same restriction-methylation enzyme pairs [38, 39]. The fact that the uptake of exogenous DNA is regulated by bacteria suggests that the process is adaptive, or at least not maladaptive.

The implication of widespread HGT-induced genetic diversity is revelatory on two levels: (1) it disproves the thesis that, compared with MCEs, prokaryotic populations of invariant clones are evolutionary sluggards; and (2) it rules out the possibility of a single, true, whole-genome phylogenetic tree for any prokaryote or its pan-genome. Rather, a prokaryote’s core-genome may have one tree, while each and every component of its flex-genome will have an independent tree. Some HGT occurs even for genes in the core-genome, meaning that in principle every gene in a prokaryotic genome could have its own evolutionary history.

### Species concepts

As we discuss below, the notion of species as it is applied to prokaryotes is substantially different from the species concept as applied to MCEs, probably to the point of being incommensurate. But, one might argue, why should this matter? There is a vast literature on “the species problem” even as applied to MCEs, yet biological research continues to advance, despite the misgivings of philosophers.

In the context of biodiversity studies (the origin of this paper), incommensurate species concepts do matter, because (a) biodiversity is a field that depends on the ability to compare and integrate biodiversity data across myriad systems—indeed, across the entire biosphere, and (b) *species* are the currency with which biodiversity is measured [40–42]. As such, these demands place constraints on species concepts, if “species” are to be useful across biodiversity science:

- To the extent that our interest in biodiversity involves the past of the biosphere (e.g., evolutionary history), we need a species definition that involves populations with shared evolutionary histories, and that (given our understanding of the way genetic material flows in MCE populations) allows the assembly of sensible trees, and ultimately, perhaps, the one true tree of life.
- To the extent that our interest in biodiversity involves the current functioning of the biosphere (e.g., community ecology), we need a species definition that correlates sensibly with roles in ecosystem dynamics and ecosystem services.
- To the extent that our interest in biodiversity is practical (e.g., the identification of organisms of economic consequence, either as sources of useful products or as pathogens), we need a species definition that correlates with genetic—and thus physiological and phenotypic—diversity.
- To the extent that our interest in biodiversity involves the future of the biosphere (e.g., conservation), we need a species definition that involves populations with shared evolutionary fates. Indeed, the whole notion of *endangered species*—a central concept in biodiversity—depends upon the idea of shared evolutionary fate.

In classical biology, individuals are seen to co-exist in local groups called *populations* and the set of all populations containing members capable of interbreeding is defined as a *species* by Mayr’s *biological species concept* (BSC). Ernst Mayr was one of the chief architects of the Modern Synthesis, the conceptual union of Neo-Darwinism (Darwin’s natural selection, augmented with Weismann’s Germ Plasm Theory) and classical genetics (i.e., genetics ideas that were post-Mendel, but pre-DNA). Mayr [43] claimed that his BSC was central to understanding biology:

BSC: Species are groups of interbreeding natural populations that are reproductively isolated from other such groups.

The species is the principal unit of evolution and it is impossible to write about evolution, and indeed about almost any aspect of the philosophy of biology, without having a sound understanding of the meaning of biological species. … The term ‘species’ refers to a *concrete phenomenon of nature* (emphasis added) and this fact severely constrains the number and kinds of possible definitions. … The BSC is based on the recognition of properties of populations. It depends on the fact of non-interbreeding with other populations. For this reason the concept is not applicable to organisms which do not form sexual populations. The supporters of the BSC therefore agree with their critics that the BSC does not apply to asexual (uniparental) organisms.

Besides the BSC, several other species concepts have been suggested, with many focused on the role and place of species in evolution and phylogenies. All scientific species concepts strive for *naturalism*—that is, for a classification that derives from some biologically causal mechanism that is intrinsic to the organisms being classified. In a review of many species concepts, Wilkins [44] noted that, “In the context of modern biology, and in particular evolutionary theory, species exist as terminal taxa in the tree of life,” and he offered a cladistic analysis to yield a roll-up description of the notion of species: “*A species is a lineage separated from other lineages by causal differences in synapomorphies*” (italics in the original). As justification, he asserted that cladistic taxa have the advantage of not requiring “some prior theoretical model; they are formed by aggregating empirical types of organisms and restricting the resulting groups to proper sets and subsets.” Here, Wilkins used “empirical types of organisms” to mean aggregations defined by lineage and he went on to claim that

[A]ny “natural” species concept involves lineal descendency and the derivation of populations from prior contiguous populations. An evolutionary understanding of species modes makes lineages fundamental and prior to species.

With the assertion that lineages are fundamental and logically prior to species, Wilkins unwittingly admits that cladistics does require a prior theoretical model, *viz*., the theoretical model of transmission genetics: that meaningful transfer of genetic information occurs—and only occurs—in a linear pattern from parent to progeny. This, again, is an assumption that only applies to MCEs.

Others, using approaches less explicitly cladistic, have also tried to find common ground among different species concepts, but the analyses always involve the assumption of linear transfer of genetic material from parents to progeny:

[A]ll contemporary species concepts share a common element and, equally important, that shared element is fundamental to the way in which species are conceptualized. The general concept to which I refer equates species with separately evolving metapopulation lineages, or more specifically, with segments of such lineages. To clarify, here the term lineage refers to an ancestor-descendant series … in this case of metapopulations or simply a metapopulation extended through time.... It is not to be confused with a clade or monophyletic group, which is sometimes also called a lineage but is generally made up of several lineages (separate branches) [45].

Hey [46] argued that much of the debate over species is an unnecessary consequence of conflating the problems of devising criteria for species identification with the more theoretical notion the way species exist in nature:

Certainly, biologists are pluralistic if we really do have different basic conceptions of species (different ideas on fundamental aspects of species existence). But, what if much of the species concept debate is actually over criteria for identifying species, and is not so much a debate over basic theoretical ideas on the causes and existence of species?

What if, Hey asks rhetorically, we just recognize that, theoretically, evolution separates organisms into groups with a common evolutionary history and that an extant species is just “the contemporaneous tip of an evolutionary lineage” [46]. Similarly, but more formally, Ereshefsky [41] noted:

[A] distinction should be made. The term “species” refers to two types of entities: species taxa and the species category. Species taxa are groups of organisms. Dog is a species taxon and chickadee is another. The species category, on the other hand, is the class of all species taxa. Our concern is with a definition of the species category: what do all species taxa have in common such that they are members of the species category?

The key to Hey’s and de Queiroz’ and Ereshefsky’s and, indeed, all attempts to define a *natural* MCE species concept (category), is the idea that the species concept (category) should be defined so that it relates to some objectively real way that organisms exist in nature. For MCEs, all such efforts have ultimately devolved into the notion of *lineage*—the idea that genetic novelty, the raw material of evolution, is acquired only once, as it is passed linearly from parents to progeny. If this is true, then in principle, one could (with perfect knowledge) track the actual flow of the genetic material through populations over time and identify all true lineages, the end points of which would be species. All members of a species would share a common evolutionary history and fate, and because of that shared history, also share similar enough genetic material that they exhibit similar attributes, both in terms of phenotype and ecological role.

Thus, *all* of the lineage-based species concepts applied to MCEs satisfy *all* of the biodiversity constraints noted above, *viz*., they are all completely anchored in the idea of shared evolutionary history and fate and they all, in consequence, deliver species groups whose members exhibit great similarity of phenotype and ecological role.

However, *none* of the species concepts as applied to prokaryotes satisfy *any* of those conditions. Paraphrasing Hey, the problems involve both the theoretical understanding of how prokaryotes exist in nature and the operational methods for identifying prokaryotic species.

The occurrence of large-scale HGT certainly has the potential to disrupt the notion of lineages, shared evolutionary fate, or shared evolutionary history, three of the key constraints on species concepts useful for biodiversity studies. Although Woese’s early work using rRNA sequences to infer prokaryotic phylogenies initially held out the promise of including prokaryotes into the tree of life, later findings about the widespread occurrence of horizontal gene transfer caused Woese [47] to doubt the universal applicability of lineage-based evolutionary analyses:

HGT is one of two keys to understanding cellular evolution. The phenomenon has long been known, but the HGT we thought we knew is not the HGT that genomics reveals. Only a decade ago HGT was generally considered a relatively benign force, which had sporadic and restricted evolutionary impact. However, the HGT that genomics reveals is not of this nature. It would seem to have the capacity to affect the entire genome, and given enough time could, therefore, completely erase an organismal genealogical trace. This is an evolutionary force to be reckoned with, comparable in power and consequence to classical vertical evolutionary mechanisms. … Yet a new realization comes with this finding: although organisms do have a genealogy-defining core of genes whose common history dates back to the root of the universal tree, that core is very small. Our classically motivated notion had been that the genealogy of an organism is reflected in the common history of the majority of its genes. What does it mean, then, to speak of an organismal genealogy when nearly all of the genes in the cell—genes that give it its general character—do not share a common history?

From both a mechanical and a theoretical perspective, the way genetic material is transmitted through organisms over time is fundamentally different in prokaryotes. This leads prokaryotic biologists to use the word “species” in a manner that is decidedly at variance with the concept as applied to MCEs. For example, in a population-genetics study on the evolution of the pan-genome of *Streptococcus* species, Muzzi and Donati [48] concluded:

Genetic exchange with related species sharing the same ecological niche was the main mechanism of evolution of *S. pneumoniae*; and *S. mitis* was the main reservoir of genetic diversity of *S. pneumoniae*.

That is, they conclude that *inter*-specific genetic exchange was a major evolutionary driver and that one species (*S. mitis*) provided the bulk of genetic variation for another species (*S. pneumoniae*). A theoretical species concept that is consistent with these assertions is incompatible with species concepts based on shared evolutionary histories and fates, the capability of interbreeding, or the contemporaneous tip of an evolutionary lineage.

This is not an atypical finding. In prokaryotes, the bulk of new gene families arise via inter-species HGT, not intra-species gene duplication [49]:

Gene duplication followed by neo- or sub-functionalization deeply impacts the evolution of protein families and is regarded as the main source of adaptive functional novelty in eukaryotes. While there is ample evidence of adaptive gene duplication in prokaryotes, it is not clear whether duplication outweighs the contribution of horizontal gene transfer in the expansion of protein families. We analyzed closely related prokaryote strains or species with small genomes (*Helicobacter*, *Neisseria*, *Streptococcus*, *Sulfolobus*), average-sized genomes (*Bacillus*, *Enterobacteriaceae*), and large genomes (*Pseudomonas*, *Bradyrhizobiaceae*) to untangle the effects of duplication and horizontal transfer. After removing the effects of transposable elements and phages, we show that the vast majority of expansions of protein families are due to transfer, even among large genomes.

With HGT providing the bulk of prokaryotic genetic novelty over time, any theoretical notion of the way prokaryotic species exist in nature cannot include the assumption that they represent the contemporary end-point of an evolutionarily isolated lineage. Whatever prokaryotic species are, they are certainly not aggregations of organisms that share a common evolutionary history or face a common evolutionary fate.

The operational methods used to identify prokaryotic species also lead to incompatibilities with MCE-oriented thinking. While early efforts to classify bacteria were largely phenomenological, a multi-factorial classification approach, named *polyphasic taxonomy*, began to emerge from a numerical approach to taxonomy [50, 51]. As Colwell [50] stated

Recent developments in biochemistry, molecular biology, and the computer sciences have intensified the already strong and fundamental interest in identifying, describing, and naming bacterial groups. It has become apparent that the new avenues of research all provide useful and meaningful data. Thus, a taxonomy is required which assembles and assimilates the many levels of information, from the molecular to the ecological, and incorporates the several distinct, and separable, portions of information extractable from a nonhomogeneous system to yield a multidimensional taxonomy. Such a taxonomy has been termed “polyphasic”.

Initially, the pioneering work by Carl Woese [52], using sequence analysis of 16S ribosomal RNA genes, provided hope that molecular analyses could provide for a true phylogenetic classification of bacteria. An effort to reconcile traditional and polyphasic prokaryotic systematics with newer molecular tools led to a report that offered a formal, molecular definition of bacterial species [53], reaffirmed in Stackebrandt et al. [54]:

The phylogenetic definition of a species generally would include strains with approximately 70 % or greater DNA-DNA relatedness and with 5 °C or less Δ*T*_*m*_.

This was augmented to include 97 % identity of 16S rRNA sequence [55], which, more recently, was raised to 98.7 % [56].

Staley [57] pointed out that this more or less arbitrary molecular threshold gives a species concept at variance with the species concept as applied to MCEs:

A major distinction between microorganisms and plants and animals concerns the definition of a species. For example, the currently accepted species definition of bacteria is based on DNA-DNA reassociation. Strains that exhibit at least 70 % reassociation by this procedure are regarded as members of the same species. This is a much broader definition of a species than that used for primates, which, like that for most plants and animals, has been based on phenotypic features and ability to interbreed. …

This arbitrary species concept is derived, in part, from the differences in genetic makeup of bacteria when compared to eukaryotic organisms. In contrast to those of eukaryotes, bacterial genomes are smaller and they are also haploid. Genetic features can be transferred among quite distantly related bacteria via various genetic exchange mechanisms such as transformation and conjugation. Genetic features may reside in the cell on a plasmid or become incorporated into the bacterial chromosome. Genetically exchanged features can be rather remarkable and have a major impact on the characteristics of the bacteria that acquire them. For example, some pathogenic species, such as *Bacillus anthracis* and *Corynebacterium diphtheriae*, are differentiated from nonpathogenic species and strains only by virtue of their plasmid-born virulence factors.

Although the application of an operational test, such as 70 % DNA-DNA hybridization, can be used to sort prokaryotes into groups that are useful for some purposes, such an operational methodology can also yield results that are logically problematic, if the notion of species is supposed to correspond to some real and natural grouping. For example, illogical results could include:

- ***Intransitivity***. Strains A and B show 74 % DNA-DNA reassociation and strains B and C show 72 % reassociation, but strains A and C show 68 % reassociation. That would make A and B members of the same species, B and C members of the same species, but A and C not members of the same species. This is somewhat analogous to the ring-species phenomenon among multi-cellular eukaryotes [58], but is more common among prokaryotes and less amenable to a natural explanation.
- ***Inconsistency***. Two bacterial strains show 69 % DNA-DNA reassociation when first measured, but 71 % reassociation after both are experimentally induced to pick up a large plasmid. The reverse would also be possible.
- ***Asymmetry***. DNA-DNA reassociation measures how much of Strain A's genome will pair with that of strain B, and vice versa. Note that, depending on the experimental protocol, the test could exhibit asymmetry, so that if there are significant size differences between the two genomes, it might be possible to find that 74 % of A reassociates to B, but only 68 % of B reassociates to A. This would mean that A is the same species as B, but B is not the same species as A.

Furthermore, the resolution, i.e., the degree of lumping vs. splitting, of a species, by virtue of the species definition employed, has profound effects upon measurements of biodiversity. For example, Staley [57] noted that different species concepts can affect whether a “species” is considered endangered (a key concept in some biodiversity contexts):

If we apply the bacterial species definition (i.e., greater than 70 % DNA-DNA hybridization) to primates, then all primates … would comprise a single species—in short, there would be only one cosmopolitan species. Furthermore, with a large population size of humans on earth, one would conclude that none of Earth’s primates are currently endangered.

Of course, the notion of *endangered species* depends upon a definition of species that includes the expectation of a unique, shared evolutionary fate across its members, an expectation that we have seen does not apply to prokaryotic species. Also, an MCE species is considered endangered when its population size drops to the point at which the possibility of collapse becomes high. However, using population size as the “endangered” threshold in prokaryotes is meaningless, because local populations are often so vast that a bucket of soil or seawater might well contain more prokaryotes than there are mammals in Africa. This challenge is further complicated by the facts that: (1) under adverse conditions many prokaryotes can enter a dormant phase, becoming essentially metabolically inactive until ambient conditions are more benign, at which time the dormant species can, within days, go from being undetectable to being the dominant member of its ecosystem; and (2) under ideal conditions a single prokaryotic cell can, within days, if not hours, generate a population of descendants numbering in the billions.

At a conceptual level prokaryotic species definitions do not equate to groups with common evolutionary trajectories, whereas the operational methods used to delineate prokaryotic species (e.g., 70 % DNA-DNA hybridization) create groups so broad that they fail to constrain group membership to organisms exhibiting highly similar phenotypes or ecological roles. As sequencing becomes increasingly cost effective, efforts continue to develop better polyphasic taxonomic methods. For example, Varghese et al. [59] employed a combination of genome-wide Average Nucleotide Identity (gANI), as well as the alignment fraction (AF) between two genomes, to measure genomic relatedness. Although such refinements will increase the subtlety of the polyphasic approach to taxonomy, it still leaves an operational definition of a prokaryote “species” that is incongruent with that employed for MCEs.

The upshot is that the MCE “species-as-lineage”—or any phylogeny-based species concept—cannot be applied to prokaryotes because they lack discrete, *whole-genome* lineages. Similarly, neither can the MCE notion of an “individual” be applied straightforwardly to prokaryotes. Efforts to squeeze prokaryotes into these MCE notions will produce results that are either metaphorical or misleading or wrong. Simply put, there is nothing in the prokaryotic realm that corresponds unambiguously to the classical ideas of individual or species.

At present, it is impossible to integrate assessments of prokaryotic biodiversity with those made of MCEs. In an essay specifically dealing with the measurement of prokaryotic biodiversity, Øvreås and Curtis [60] asserted that

Traditional biodiversity is based on the “species” as a unit. In microbial ecology the species concept is useless as the species concept for bacteria is obscure.

They go on to note that the diversity in microbial ecosystems is so vast (10^4^ to 10^6^ taxa in a single gram) that, so far, it has been practically impossible to develop sampling methods appropriate for measuring this diversity: “Sample sizes are still dictated by what is feasible, not what is required” ([60], p. 224). They conclude that new methods and new understandings will need to be developed if the diversity of the prokaryotic realm is to be documented and understood:

We need to create a new generation of numerate and computer savvy molecular microbial ecologists to explore this immense frontier. They will no doubt regard much of what has been done in the past 30 years as quaint and primitive.

### Reconsidering the MCE individual

Classical biology is anchored by the concept of the individual organism. In traditional thinking about evolution, biodiversity, and ecosystems, the concept of “individual organism” occupies a position as fundamental as “gene” was to classical genetics. In *Principles of Animal Taxonomy*, George Gaylord Simpson [61] wrote, “It seems obvious … that the real unit in nature, the one thing that is usually completely objective in spite of some marginal cases, is the individual organism.” This thinking infused the development of the Modern Synthesis.

Traditionally, an MCE individual was seen as a physically coherent aggregation of cells, all clonally derived from a single cell and all receiving its DNA only from its immediate ancestor(s). The cells of these individuals undergo differentiation into germ-line cells (ultimately gametes) and somatic cells—a “body” of tissues and organs to protect and nurture the germ line, at least until reproduction. To do this, all of the cells must cooperate with each other in a highly controlled and regulated manner, in turn requiring an extremely stable, shared and identical (nearly) genome. Ultimately, in MCEs all somatic cells live or die together as a single individual entity. In the modern synthesis, the differential survival of somatic-cell individuals and their genetically identical germ-line payloads is the driving mechanism of evolution.

Given the centrality of “the individual” to MCE thinking, it is not surprising that much of classical biology is dedicated to studying the attributes and behavior of aggregations of somatic cells, i.e., individual *organisms*. Primatology, for example, is largely the study of the structure, behavior, and physiology of primate somatic cells. When classical biology does treat germ-line cells, it is often in terms of gamete production and fertilization—that is, the germ line is viewed from the perspective of the soma.

Prokaryotes, with their non-mechanical relationship to the environment, their non-reproductive gene acquisition (HGT), their lack of enforced stability of genomic content, and their lack of differentiated somatic tissue, are the antithesis of MCE organismal individuality. Of course prokaryotes can, and do, exhibit individuality on the cellular level. But, critically, an individual prokaryotic cell is free from the requirement of maintaining genomic identity with neighboring cells and needs only to maintain its basic viability long enough to carry out basic physiological functions, transfer or acquire genes, replicate its genome, and divide. Consequently, prokaryotes, unlike MCEs, have the great advantage of being able to acquire or discard genes for almost any non-critical function without the penalty of adversely affecting somatic development or regulation. Essentially, a prokaryote functions as one cell interacting physiologically with its environment, and with genetic content that can vary independently of reproduction.

More profoundly, new research on microbiomes (microbial communities that are physically engaged with multicellular organisms) are forcing a re-evaluation of the notion of individuality, even among MCEs. It has long been known that every MCE individual carries huge numbers of microbes on every available surface, in every orifice, and sometimes endosymbiotically within cells. Recent studies have demonstrated that these associated microbiomes often play essential roles in the normal physiology and function of the host, contributing positively to the host’s fitness and affecting how it interacts with its environment [62].

If associated microbiomes affect the fitness of the MCE host, then even among MCEs the primary unit of evolutionary survival and ecological function is not Simpson’s “objectively real” individual, but rather the *holobiont*—the composite of one MCE organism and its associated microbiome communities [63–66]. Some have asserted the conceptual demise of the individual with the suggestion that, “We are all lichens now” [62, 67].

It is no longer possible to claim that individual organisms are objectively real, fundamental units in nature. Instead, we must now recognize that the classical concept of “individual” is, at best, a reductionist abstraction, in the way “assume a spherical cow” is useful in biophysics—it simplifies the analysis, but at some cost to a correspondence with reality.

### The tree(s) of life

As fundamental units of evolution, individual organisms are held to be evolutionarily related within species and higher clades that, in turn, compose a single-rooted tree of life. But genomics, particularly the metagenomics of biological dark matter, reveals these truths to be, again, useful approximations restricted to the MCE realm. In fact, just as there are no “completely objective” individuals, there is no one true tree of all of life on Earth.

**Fig. 2:**
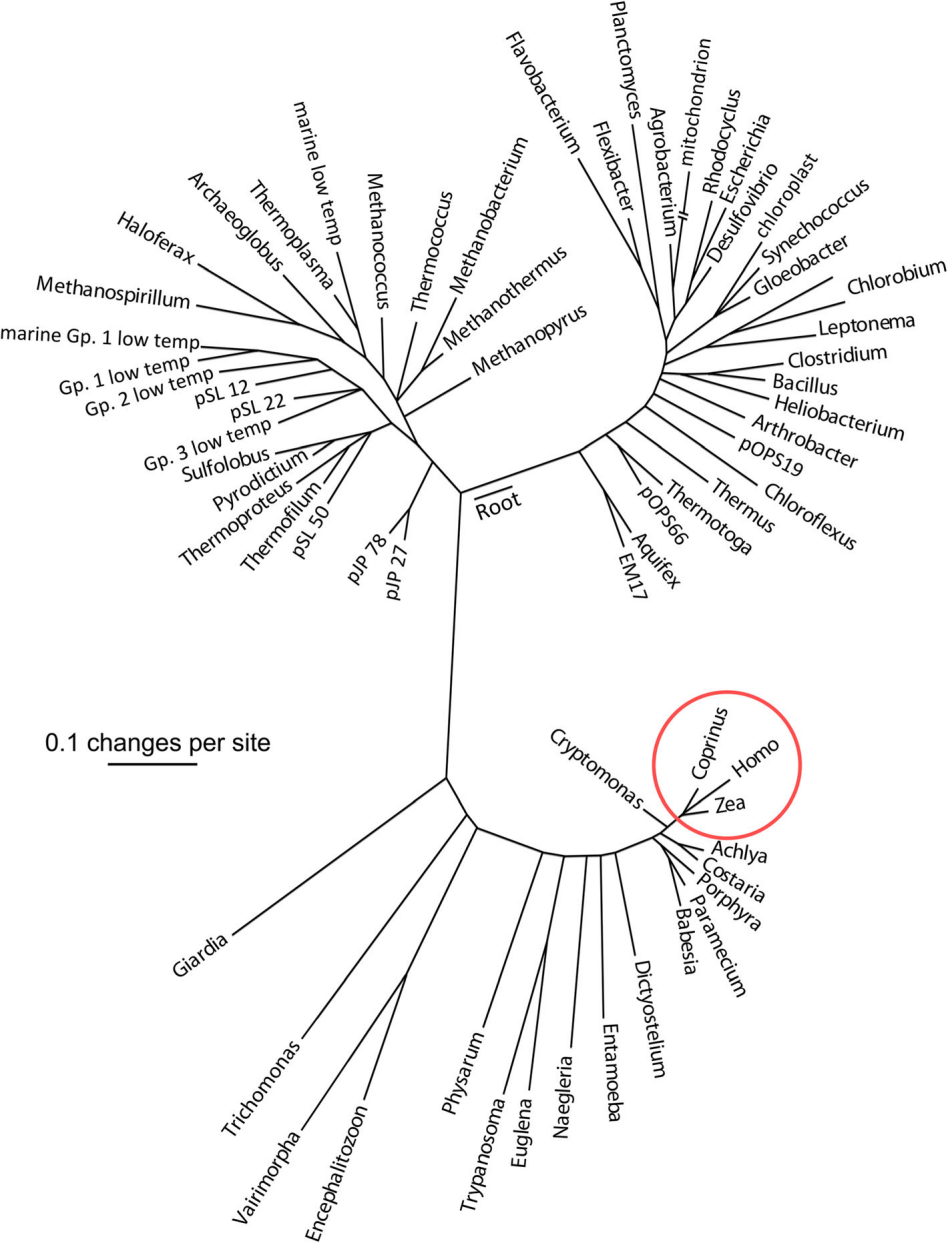
The evolutionary relationships across all three domains of life, as reflected in the sequence similarity of their small subunit rRNA genes (after [68]). The differences among all the MCEs (inside the circle) is trivial, compared with the rest of the biosphere

By the late 1990s, routine sequencing technology could use full, rather than indirect, measures of rRNA to construct evolutionary relationships among *all* life forms on Earth [68], or at least among their rRNA genes. In this universal tree (Fig. 2), with branch lengths proportional to rRNA sequence differences, all MCEs—animals, plants, and fungi—are encompassed within the small circle. From this perspective, all MCEs are a highly differentiated, specialized, and atypical form of life, no more representative of the entire biosphere than, say, hummingbirds are of the vertebrates.

Woese [69], citing the implications of horizontal gene transfer for the concept of a tree of life, was quick to dismiss old notions.

Classical biology has also saddled us with a phylogenetic tree, an image the biologist invests with a deep and totally unwarranted significance. The tree is no more than a representational device, but to the biologist it is some God-given truth. Thus, for example, we agonize over how the tree can accommodate horizontal gene transfer events, when it should simply be a matter of when (and to what extent) the evolution course can be usefully represented by a tree diagram: Evolution defines the tree, not the reverse.

As discussed above, rampant horizontal gene flow among prokaryotes falsifies the tenet that the evolutionary history of all organisms—and their genes—can be reflected unambiguously by a single, tree-like pattern. It is important to remember that Pace’s universal tree (Fig. 2) is a tree of small subunit rRNA genes, not a tree of *life*.

### Population dynamics

The MCE concepts of “species” and “individual” are deeply embedded in most efforts to characterize and measure biodiversity. Most MCE diversity metrics are defined in terms of “species diversity” over some spatio-temporal range, and much raw biodiversity-measurement data consists of observations of the occurrence of a single individual of a particular species at a specific point in space and time. As shown above, MCE-oriented concepts of species and individual cannot be applied meaningfully to prokaryotes. Further, the case can be made that prokaryote populations operate on such incomparably different spatio-temporal scales that it is difficult to describe a “specific point in space and time” in a way that applies equally effectively to MCE and to prokaryote populations.

If one were to go into any MCE ecosystem, pick a species at random, kill off 99.9 % of its population, and then measure how long it would take the species to recover, the result might be a year or more for a mouse, and a century or more (if then) for elephants. For some prokaryotes, the time to recover from a 99.9 % population cull could be 24 h or less. From a biodiversity perspective this means that, in principle, a rare prokaryotic species (say, 0.1 % of the population) could become the dominant species (say, 90 % of the population) in a very short period of time. Some recent reports suggest that this does in fact occur in natural populations [70–72].

Shade et al. [72] used “16S rRNA amplicon sequencing of 3237 samples from 42 time series of microbial communities from nine different ecosystems (air; marine; lake; stream; adult human skin, tongue, and gut; infant gut; and brewery wastewater treatment)” to examine significant changes in microbial community composition that occur when typically rare taxa become very abundant, either in response to disturbance or periodic change in the environment. They designated such taxa as “conditionally rare taxa” or CRT. They discovered that

CRT made up 1.5 to 28 % of the community membership, represented a broad diversity of bacterial and archaeal lineages, and explained large amounts of temporal community dissimilarity (*i.e*., up to 97 % of Bray-Curtis dissimilarity). Most of the CRT were detected at multiple time points, though we also identified “one-hit wonder” CRT that were observed at only one time point. Using a case study from a temperate lake, we gained additional insights into the ecology of CRT by comparing routine community time series to large disturbance events. Our results reveal that many rare taxa contribute a greater amount to microbial community dynamics than is apparent from their low proportional abundances. This observation was true across a wide range of ecosystems, indicating that these rare taxa are essential for understanding community changes over time.

Similarly, Aanderud et al. [70] investigated the effect of rewetting upon dry soil samples taken from various ecosystems. They defined as “rare” any species that could not be detected in the dry sample but could be detected in the wetted sample. They found that, across all ecosystems, “rewetting had strong effects on bacterial community composition”. In their metagenomic samples from rewetted environments:

Rare bacteria comprised 69-74 % of taxa and nearly 60 % of the 16 s rRNA gene sequences in rewetted communities, irrespective of the ecosystem sampled. … This rapid turnover of the bacterial community corresponded with a 5–20-fold increase in the net production of CO_2_ and up to a 150 % reduction in the net production of CH_4_ from rewetted soils. Results from our study demonstrate that the rare biosphere may account for a large and dynamic fraction of a (microbial) community.

In a review, Shade and Gilbert [71] noted that although CRTs are, by definition, usually rare in microbial communities, they account for 97 % of temporal variability in microbial community structure. They note that microbial community ecology cannot be understood without recognizing the dynamic nature of microbial ecosystem community structure:

Accounting for the dynamic patterns of rare taxa will only improve our understanding of the ecology of microbial communities. Recognizing that many, if not most, members of microbial communities exhibit abundance changes over time will help to move microbial community ecology from static laundry lists of taxa to dynamic models that will allow us to better predict, manage, and remodel microbial consortia.

Taken together, these findings suggest that microbial ecosystems may routinely and rapidly undergo profound changes in community structure that, for MCEs, would be described as a major ecological succession. Among MCEs, however, a major ecosystem succession may take years, decades, or even centuries, whereas dynamic changes in microbial communities may well occur over hours or days.

Clearly, time-series measurements that would adequately characterize an MCE ecosystem community would be completely inadequate (i.e., off by several orders of magnitude) for capturing and characterizing the dynamic nature of a microbial ecosystem. If global measures of biodiversity are to include prokaryotic communities, substantial work will have to be done to determine the appropriate time-scale for capturing their real attributes.

The problem of appropriate measurement scale also applies to space. MCE biodiversity is often assessed by sampling the environment along a transect or on some grid. But what would be the appropriate sampling grid for assessing, say, prokaryotic biodiversity in soil? Scaled by body size, assessing soil communities with samples taken five meters apart would be equivalent to assessing mouse biodiversity with samples taken forty miles apart or elephant biodiversity with samples three thousand miles apart.

Before samples of prokaryotic biodiversity can be effectively included in global assessments of biodiversity, substantial work must be done to determine optimal spatio-temporal scales for sampling [60].

## Conclusion: biological dark matter and quantum biology

Biology is no stranger to paradigm shifts. Before the advent of molecular biology, classical genetics was based on the notions that genes are the fundamental units of mutation, of recombination, and of heredity, and that they are arranged on the chromosomes like beads on a string. New insights, generated by molecular analysis, forced a recognition that there were, in fact, no fundamental units, no beads, and no string.

Similarly, before metagenomic tools allowed us to see the prokaryotic world, classical biology held that individual organisms are the fundamental units of ecology, of evolution, and of biodiversity, and their evolutionary history could be explained by arranging them into objectively real, lineage-defined groups, with the groups composed into a single-rooted tree. New insights from molecular analysis are forcing a recognition that there are, in fact, no completely objective individuals, no unique lineages, and no one true tree, at least in the quantum realm of prokaryotic dark matter.

Although many biologists still think of the biosphere primarily in terms of MCEs (despite their demonstrable atypicality), the flood of revelations from metagenomics will ultimately force a change. We now know that about half of the world’s biomass and by far the majority of its genetic biodiversity actually occur as prokaryotic microbial communities. We also know that the visible biosphere of MCE macrobiological life represents a highly derived and constrained subset of life on Earth. Most importantly, we know that *every* individual of the MCE biosphere is completely covered and infused with prokaryotic life and that these associated microbiomes have significant effects on the function and fitness of their MCE hosts.

In biology, as in physics, macroscale-level properties (i.e., those of classical biology) do not apply at the level of the micro-scale (i.e., biological dark matter). The newly emerging biosphere of microbial dark matter cannot be made tractable to research or understood merely by extending the concepts and methods of eukaryotic macrobiology. By analogy, that would prove about as useful as attempting to understand quantum mechanics through the application of Newtonian mechanics to miniature billiard balls.

The unveiling of biological dark matter is allowing us to see, for the first time, the diversity of the entire biosphere and, to paraphrase Darwin, is providing a new view of life. Advancing and understanding that view will require major revisions to some of the most fundamental concepts and theories in biology.

## Abbreviations

BSC: Biological species concept
CRT: Conditionally rare taxa
HGT: Horizontal gene transfer
MCE: Multicellular eukaryote
